# Photocatalytic Degradation of Tobacco Tar Using CsPbBr3 Quantum Dots Modified Bi2WO6 Composite Photocatalyst

**DOI:** 10.3390/nano11092422

**Published:** 2021-09-17

**Authors:** Runda Huang, Menglong Zhang, Zhaoqiang Zheng, Kunqiang Wang, Xiao Liu, Qizan Chen, Dongxiang Luo

**Affiliations:** 1 School of Materials and Energy, Guangdong University of Technology, Guangzhou 510006, China; 2111902118@mail2.gdut.edu.cn (R.H.); zhengzhq5@mail2.sysu.edu.cn (Z.Z.); qizanchen@gdut.edu.cn (Q.C.); 2 Institute of Semiconductors, South China Normal University, Guangzhou 510631, China; 3 School of Chemistry and Chemical Engineering, Institute of Clean Energy and Materials, Guangzhou Key Laboratory for Clean Energy and Materials, Huangpu Hydrogen Innovation Center, Guangzhou University, Guangzhou 510006, China; kunqiangwang@m.scun.edu.cn

**Keywords:** nanocomposites, photocatalytic degradation, perovskite, tobacco tar

## Abstract

Polycyclic aromatic hydrocarbons (PAHs) in tobacco tar are regarded as a significant threat to human health. PAHs are formed due to the incomplete combustion of organics in tobacco and cigarette paper. Herein, for the first time, we extended the application of CsPbBr_3_ quantum dots (CsPbBr_3_) to the photocatalytic degradation of tobacco tar, which was collected from used cigarette filters. To optimize the photoactivity, CsPbBr_3_ was coupled with Bi_2_WO_6_ for the construction of a type-II photocatalyst. The photocatalytic performance of the CsPbBr_3_/Bi_2_WO_6_ composite was evaluated by the degradation rate of PAHs from tobacco tar under simulated solar irradiation. The results revealed that CsPbBr_3_/Bi_2_WO_6_ possesses a large specific surface area, outstanding absorption ability, good light absorption and rapid charge separation. As a result, in addition to good stability, the composite photocatalyst performed remarkably well in degrading PAHs (over 96% were removed in 50 mins of irradiation by AM 1.5 G). This study sheds light on promising novel applications of halide perovskite.

## 1. Introduction

The development of economies can create problems such as energy shortages and environmental pollution, which are regarded as critical challenges to global sustainable development. In recent years, industrial and environmental pollution caused by organic pollutants has attracted considerable public attention [1,2]. In terms of human health, the main concerns include benzene compounds [3], formaldehyde [4], bacterial infections [5], and tobacco tar [6]. In particular, it is well known that tobacco tar is formed due to the incomplete combustion of organics in tobacco and cigarette paper. The substrates of tobacco are very complicated, and among these organic substrates, polycyclic aromatic hydrocarbons (PAHs) are considered as the major pollutants. This can be attributed to their bio-accumulative, toxic, carcinogenic, and recalcitrant features [7]. Thus, minimizing the toxicity of tobacco tar is desirable for human health and the environment. However, until now, not much research has been carried out on the degradation of tobacco tar via photocatalysis.

Among the techniques used for organic pollutant degradation, photocatalytic oxidation uses low-cost semiconductive materials and is considered as an environmentally-friendly path for the purification or transformation of organic pollutants [8,9,10,11]. Semiconductor-based photocatalytic reactions have already attracted extensive attention because they are a simple and environmentally friendly way to utilize solar energy. Therefore, numerous types of photocatalysts have been extensively investigated, such as TiO_2_, ZnO, SnO_2_, SrTiO_3_, LaFeO_3_, BaTiO_3_, and so on [12,13,14,15,16,17]. However, these semiconductors still have some common shortcomings, such as a narrow light absorption range and a high recombination ratio of photogenerated charges [18]. In this case, there is an urgent need to develop visible-light driven and highly active photocatalysts.

Bi_2_WO_6_ is currently regarded as a promising photocatalyst for its outstanding photo-redox ability, nontoxicity, and thermal and chemical stability [19]. Its potential applications include solar cells, photoelectrodes and photocatalysis [20,21,22]. However, the rapid recombination of exitonic pairs, ineffective charge separation and unsatisfactory visible-light absorption have limited the photocatalytic performance of pure Bi_2_WO_6_ [23]. Hence, different strategies have been applied to improve the photocatalytic properties of Bi_2_WO_6_, such as coupling with other semiconductors, morphological control, ion doping and surface modification [24,25,26,27,28]. One of the most accessible approaches is the deposition of quantum dots (QDs) onto the semiconductor surface, which is beneficial due to their noteworthy properties, including high charge separation efficiency and quantum confinement effects [29,30]. Thus, modification with QDs is regarded as an effective method to improve the photocatalytic performance of semiconductor materials.

Among the various QDs materials, CsPbBr_3_ QDs have received increasing interest due to their high luminescent quantum yields [31] in solid states in the green range (band gap energy = 2.4 eV) and superior environmental stability in comparison to other perovskites with organic cations [32]. Perovskite materials possess unique advantages in regard to effective charge separation and electron hole diffusion, which makes them suitable not only for the photovoltaic field, but also for several photocatalytic reaction types [33,34]. In addition, CsPbBr_3_ is very attractive due to its high thermal-stability and photo-resistance [35]. Considering these advantages, CsPbBr_3_ was used to decorate Bi_2_WO_6_ via a hydrothermal method in this work and the photoactivity was evaluated through the photocatalytic degradation of PAHs in tobacco tar under simulated solar light. Additionally, the CsPbBr_3_/Bi_2_WO_6_ heterogeneous photocatalyst exhibited increased specific surface area and improved light absorption in the visible range, which effectively promoted the photocatalytic activity [36]. Compared with pure Bi_2_WO_6_, the CsPbBr_3_/Bi_2_WO_6_ composites exhibit superior photocatalytic activity and stability. This work presents a promising strategy for designing a more efficient composite photocatalyst-based semiconductor photocatalyst for application to pollutant purification. In addition, to the best of our knowledge, this work is the first to utilize perovskite for the degradation of PAHs.

## 2. Experimental Section

### 2.1. Reagents and Materials

Bismuth nitrate pentahydrate (Bi(NO_3_)_3_·5H_2_O, 99%), ethanol (CH_3_CH_2_OH, 99.7%), nitric acid (HNO_3_, 68%), sodium tungstate dehydrates (Na_2_WO_4_·2H_2_O, 99.5%) were purchased from Aladdin Industrial Co, Ltd. (Shanghai, China) and CsPbBr_3_ perovskite QDs was purchased from Nanjing MKNANO, China and used as received. The used cigarette filters were collected randomly. The deionized water used throughout all experiments was purified through a Millipore system (Millipore, Billerica, MA, USA).

### 2.2. Synthesis of CsPbBr_3_/Bi_2_WO_6_

Pure Bi_2_WO_6_ was synthesized via a hydrothermal method. First, Bi(NO_3_)_3_·5H_2_O (1 mmol) was dissolved in an aqueous solvent mixture of 30 mL HNO_3_ (pH = 3) and 20 mL Na_2_WO_4_·2H_2_O (0.5 mmol). The mixed solution was then vigorously stirred until clear, under room temperature. The solution was subsequently heat-treated in a muffle furnace at 120 °C for 2 h. The resultant precipitate was dried to obtain the pure Bi_2_WO_6_ photocatalyst (light-yellow powder). Subsequently, 1 mL of CsPbBr_3_ suspension (10 mg/mL, purchased from Nanjing MKNANO, Nanjing, China) was ultrasonicated for 30 min to generate CsPbBr_3_ QDs dispersion. Then, a certain amount of Bi_2_WO_6_ was added into the above CsPbBr_3_ suspension and stirred for 20 h. For example, to prepared 10 wt% CsPbBr_3_/Bi_2_WO_6_, 90 mg of Bi_2_WO_6_ was added to the 1 mL suspension. Subsequently, the mixture was evaporated at 40 °C for 10 h and the composite photocatalyst was obtained after drying.

### 2.3. Photocatalytic Experiments

The tobacco tar was obtained by immersing 10 randomly collected used cigarette filters in 50 mL trifluorotoluene, followed by 10 min ultra-sonification to give a brown-yellow tobacco tar solution. The cigarette filters were then removed from the solution. The concentrations of the substrates in the tobacco tar solution were confirmed using gas chromatography (GC). The as-prepared photocatalyst was uniformly dispersed in 2 mL of tobacco tar solution with 20 min ultrasonic treatment. Then, the samples were illuminated by Xe lamp with an AM 1.5 G filter as the simulated solar source. The photoactivity was confirmed by monitoring the concentrations of PAHs under different illumination time.

### 2.4. Characterization

The crystal structure of the samples was studied by X-ray diffractometry (XRD; Bruker-AXS D8 Advance, Karlsruhe, Germany). Field emission scanning electron microscopy (SEM) (Hitachi S-4800, Tokyo, Japan) and transmission electron microscopy (TEM) (JEOL JEM-2100, Tokyo, Japan) were performed for the morphological and microstructural analyses, respectively, of the samples. The UV-vis absorption spectroscopy of the samples was performed by a UV-vis near-infrared spectrophotometer (Agilent Cary 5000, Palo Alto, CA, USA). The XPS energy spectrum was determined by an X-ray photoelectron spectrometer (Thermo Fisher/Escalab 250Xi, Waltham, MA, USA). Measuring the surface area and pore size distribution of samples was performed using a specific surface and porosity analyzer (Micromeritics ASAP2460, Atlanta, GA, USA). TGA analysis was done by using the Mettler Toledo TGA/DSC 3+ instrument (Zurich, Switzerland). Total organic carbon (TOC) content in solvent was assessed by a TOC analyzer (Shimadzu, TOC-L-VCPN, Tokyo, Japan). The photocatalytic result was characterized by single quadrupole gas chromatography-mass spectrometry (Thermo Fisher/Trace1300 ISQ QD, Waltham, MA, USA).

## 3. Results and Discussion

### 3.1. Properties of the Composite Photocatalysts

**Figure 1:**
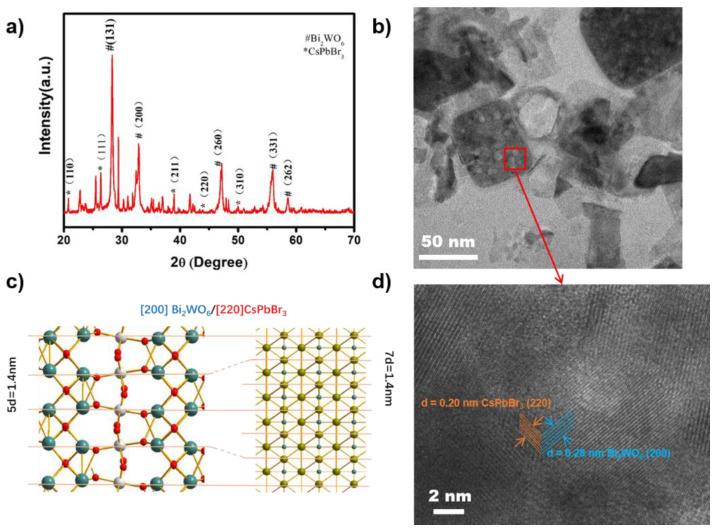
(**a**) PXRD patterns and (**b**) TEM image of the as-prepared 15 wt% CsPbBr_3_/Bi_2_WO_6_ photocatalyst; (**c**) schematic diagram of semi-coherent interface of the two components; (**d**) HR-TEM image of the as-prepared 15 wt% CsPbBr_3_/Bi_2_WO_6_ photocatalyst.

Powder X-ray diffraction (PXRD) (Figure 1a) was initially exploited to evaluate the phase and crystallinity of the as-prepared CsPbBr_3_ QDs-decorated Bi_2_WO_6_ (CsPbBr_3_/Bi_2_WO_6_). A p-FTO glass slide was used as a substrate to support the samples for characterization. The main peaks at 2θ of 28.3°, 32.8°, 47.0°, 55.8° and 58.5° can be indexed to an orthorhombic Bi_2_WO_6_ (JCPDS#39-0256), and corresponded to the indices of (131), (200), (260), (331) and (262) planes, respectively. On the other hand, the diffraction patterns of cubic CsPbBr_3_ corresponded to JCPDS#54-0752 and a previous report [32,37]. The transmission electron microscopy (TEM, Figure 1b) revealed that the CsPbBr_3_ QDs of a few nanometers were dispersed on Bi_2_WO_6_ with a quasi-rectangle morphology. The two materials were subsequently confirmed by high-resolution transmission electron microscopy (HR-TEM), which showed the lattice spacing of CsPbBr_3_ ([220] = 0.20 nm) and Bi_2_WO_6_ ([200] = 0.28) (Figure 1d), which can be compared to the diffraction results from PXRD. In addition, as illustrated in Figure 1c, the connected boundary presented as a semi-coherent interface involving several unit cells. The lattice mismatch between the crystal planes of these two components can be defined as Δd/d_Bi2WO6_ = 28%. Furthermore, as shown in Figure 1c,d, CsPbBr_3_ and Bi_2_WO_6_ exhibited periodically coincided lattice (7d (CsPbBr_3_) = 5d (Bi_2_WO_6_)), suggesting that intimate energetic and chemical interactions were formed at the semi-coherent interface of the two components [38].

**Figure 2:**
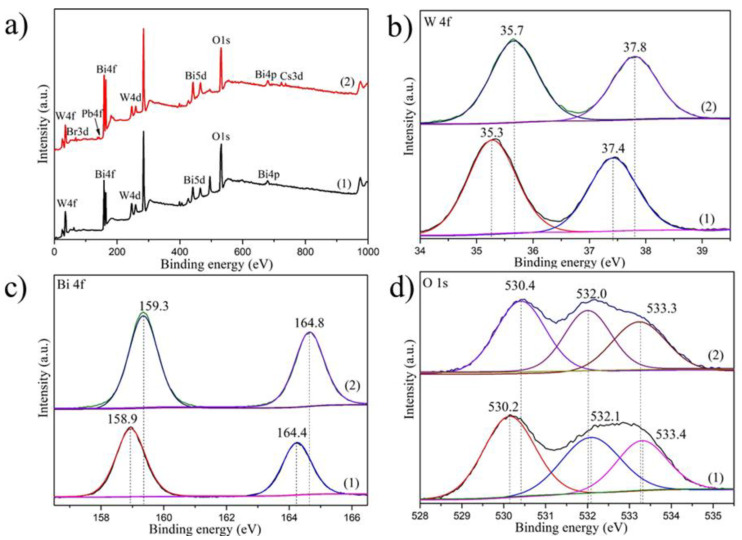
XPS spectra of (1) pure Bi_2_WO_6_ and (2) 15 wt% CsPbBr_3_/Bi_2_WO_6_ samples. (**a**) Survey scan; (**b**) W4f; (**c**) Bi4f; (**d**) O1s.

The elemental composition of the as-prepared pure Bi_2_WO_6_ and CsPbBr_3_/Bi_2_WO_6_ were confirmed by X-ray photoelectron spectroscopy (XPS), which demonstrated that the CsPbBr_3_/Bi_2_WO_6_ sample contained no other elements apart from O, Bi, Cs, Pb, Br, C 1 and W (Figure 2a). The surface atomic content was calculated by the fitting peak area and sensitivity factor, as shown in Table 1 (the carbon atomic content comes from the XPS instrument). Besides, the altered chemical state of the coupled materials was recorded by XPS as well; as shown in Figure 2b–d, the characteristic peaks of W4f_5/2_ and W4f_7/2_ in pristine CsPbBr_3_/Bi_2_WO_6_ were located at 37.8 and 35.7 eV (Figure 2b), the characteristic peaks of Bi4f_5/2_ and Bi4f_7/2_ were located at 164.8 and 159.3 eV (Figure 2c), and the characteristic peaks of O1s were located at 533.3, 532.0 and 530.4 eV (Figure 2d). In the Bi_2_WO_6_ sample, the characteristic peaks of W4f_5/2_ and W4f_7/2_ were located at 37.4 and 35.3 eV (Figure 2b), the characteristic peaks of Bi4f_5/2_ and Bi4f_7/2_ were located at 164.4 and 158.9 eV (Figure 2c), and the characteristic peak of O1s was 533.4, 532.1 and 530.2 eV (Figure 2d). This showed that the valence state of Bi, W and O in the composite sample was +3, +6 and −2, respectively. In comparison to pristine Bi_2_WO_6_, the binding energy of Bi4f, W4f in the CsPbBr_3_/Bi_2_WO_6_ samples shifted ca. 0.7 and 0.4 towards the higher energy, respectively. This could be attributed to the regional environment and electron density change of Bi_2_WO_6_ and CsPbBr_3_ in the composites due to strong heterojunction formation [39]. In detail, the Bi_2_WO_6_ displayed increased electron accepting capacity and decreased electron density. On the other hand, this is accompanied by an increase in the electron density and an improvement in the electron-donating capacity on the CsPbBr_3_ side. Consequently, the internal electric field was established, leading to a tendency for electron transfer from CsPbBr_3_ to Bi_2_WO_6_, and as a result, a typical type-II band structure was formed.

In order to explore the electronic features of CsPbBr_3_/Bi_2_WO_6_, Hall effect measurement was employed to monitor the charge carrier density and the Hall mobility. Hall effect measurement is an important approach for investigating the charge transport mechanisms in Bi_2_WO_6_ with or without the coupling of CsPbBr_3_ QDs. Here, as shown in Table 2, the Bi_2_WO_6_ sample exhibited lower charge carrier density (2.738 × 10^11^ cm^−3^) than that of CsPbBr_3_/Bi_2_WO_6_ (5.154 × 10^14^ cm^−3^). In contrast to the reduced charge density in the Z-scheme due to the recombination of electrons and holes between the two component materials, this result was consistent with the type-II feature of the composite materials, which possessed accumulated charges. Meanwhile, greater mobility was observed from pristine Bi_2_WO_6_ in comparison to that of CsPbBr_3_/Bi_2_WO_6_; this can be attributed to the effective mass of the carriers. According to the equation: $\mu =\frac{\tau e}{m}$, the mobility (*μ*) is proportional to the elemental charge (*e*) and carrier mean free time (τ). In contrast, the mobility is inversely proportional to the effective mass of the carriers (*m*), and a greater *m* typically indicates larger carrier density [40].

**Figure 3:**
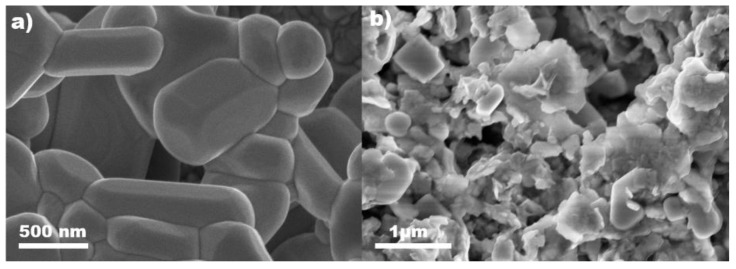
SEM images of (**a**) Bi_2_WO_6_ and (**b**) CsPbBr_3_/Bi_2_WO_6_ photocatalysts.

The morphologies of the as-prepared samples were confirmed by scanning electron microscopy (SEM). The Bi_2_WO_6_ displayed particles of a few hundred nanometers with exposed flat and smooth facets as shown in Figure 3a. In comparison to the pure Bi_2_WO_6,_ the as-prepared CsPbBr_3_/Bi_2_WO_6_ composite exhibited a rougher morphology due to the decoration of CsPbBr_3_ dots on the facets of Bi_2_WO_6_ (Figure 3b). These SEM results were consistent with the cubic-shaped particles on the micro-square observed by TEM under low magnification (Figure 1b). The rough morphologies of the composite material lead to efficient light absorption due to their light trapping effect [41]. Besides, the surface area could be increased due to the presence of CsPbBr_3_ quantum dots on Bi_2_WO_6_, which was confirmed by N_2_ adsorption-desorption isotherms. The samples presented typical Type-IV adsorption isotherms with a H_3_ hysteresis loop (Figure S1a), illustrating the porous nature of the catalysts [42]. Furthermore, the specific surface area of the Bi_2_WO_6_ and CsPbBr_3_/Bi_2_WO_6_ were compared and CsPbBr_3_/Bi_2_WO_6_ exhibited a more than 4-fold larger surface area than that of Bi_2_WO_6_. The details of the isotherm studies can be found in Table 3. The high surface area of CsPbBr_3_/Bi_2_WO_6_ suggested an improvement in the active sites and adsorption capacity, which allows rapid interaction with the reactants. Next, the pure Bi_2_WO_6_ and CsPbBr_3_/Bi_2_WO_6_ were subjected to thermal gravimetric analysis (TGA) using -temperatures ranging from room temperature to 600 °C with a heat rate of 5 °C/min in fluid He (20 mL/min). As illustrated in Figure S1b, CsPbBr_3_/Bi_2_WO_6_ exhibited improved thermal stability in comparison to the pure Bi_2_WO_6_, especially from 250 °C to 600 °C. This could be attributed to the strong interaction between CsPbBr_3_ and Bi_2_WO_6_.

**Figure 4:**
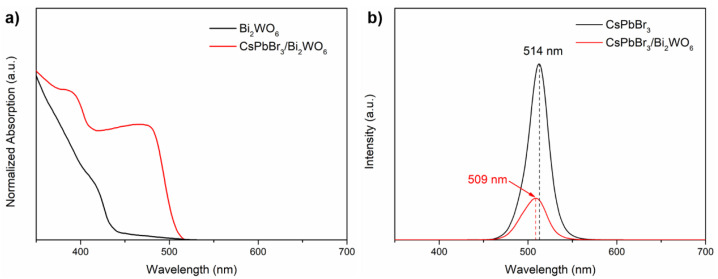
(**a**) UV-vis absorption spectra and (**b**) steady-state PL spectra of CsPbBr_3_ and 15 wt% CsPbBr_3_/Bi_2_WO_6_.

In Figure 4a, the UV-vis absorption spectra of pure Bi_2_WO_6_ and the CsPbBr_3_/Bi_2_WO_6_ composite are illustrated. The pure Bi_2_WO_6_ presented an absorption edge at ca. 445 nm, corresponding to its typical band gap energy of 2.78 eV. After loading with CsPbBr_3_ QDs, a dramatically enhanced absorption below ca. 510 nm was observed, which indicated the optimized light harvesting capacity of the composite photocatalyst [38,43,44,45]. In addition, as presented in Figure 4b, a decreased intensity and slightly blue-shift in the photoluminescence (PL) peak was observed when coupling the CsPbBr_3_ with Bi_2_WO_6_, which indicated that the recombination rate of electron-hole pairs were suppressed, and thus they promote the photocatalytic efficiency [46].

### 3.2. Activity for Photodegradation of PAHs from Tobacco Tar

Trifluorotoluene was employed as the solvent to dissolve the tobacco tar due to its apolar nature, good solubility, and superior chemical stability comparing with toluene. A list of the main substrates in the tobacco tar solution identified by gas chromatography-mass spectrometry (GC-MS) is given in Table S1. A Xe lamp (100 mW cm^−2^) coupled with AM 1.5 G filters were utilized as the simulated solar source for illumination. GC-MS and UV-visible absorption spectra (UVAS) were exploited to evaluate the products and degradation rate of the photocatalysts. A series of tests was carried out under room temperature, and the photocatalytic reactor was saturated with different matter (air, H_2_O, and O_2_) to investigate the key radicals for promoting the degradation rate. To reach adsorption equilibrium, the as-prepared photocatalysts were initially mixed with tobacco tar under stirring (1200 rpm) in the dark for 30 min prior to the photocatalytic reactions.

**Figure 5:**
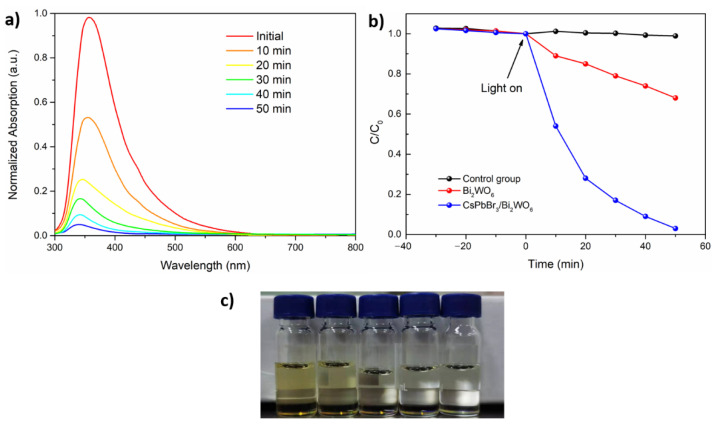
(**a**) The absorption spectra of the samples using 15 wt% CsPbBr_3_/Bi_2_WO_6_ as a photocatalyst for different times; (**b**) photocatalytic degradation of tobacco tar using pure Bi_2_WO_6_, 15 wt% CsPbBr_3_/Bi_2_WO_6_ and control group (without photocatalyst); (**c**) digital photographs of the sample using 15 wt% CsPbBr_3_/Bi_2_WO_6_ as a photocatalyst for 10 to 50 min (from left to right). Reaction condition: 50 mg photocatalyst, saturated with air, stirred in the dark for 30 min before experiments; then, the reactions were conducted under AM 1.5 G simulated light irradiation for 50 min.

As reported previously [47,48], the highly reactive hydroxyl radicals (·OH) and superoxide radicals (·O_2_^−^) formed in the photocatalytic process are desirable for degrading PAHs into less toxic products. Thus, the samples were saturated with air to supply the source H_2_O and O_2_ needed to accelerate the generation of hydroxyl and superoxide radicals. As shown in Figure 5, approximately 32% of the PAHs was degraded with the pure Bi_2_WO_6_ photocatalyst in 50 min. This can be compared to the CsPbBr_3_/Bi_2_WO_6_ composite, which presented a significantly improved degradation rate. This can be attributed to the efficient charge separation of the type-II band structure and the enhanced light absorption of the CsPbBr_3_. In addition, the degradation rate was systematically enhanced with the increasing weight ratio of the CsPbBr_3_ in the composites (Figure S2), and reached its maximum at 96% when loaded with 15 wt% CsPbBr_3_ (Figure 5a,b). The total organic carbon (TOC) content in the solvent was assessed by a TOC analyzer to further reflect the degree of PAHs degradation. As shown in Table S2, the TOC content in the reaction system decreased from 5.36 mg/L before the reaction to 2.3 mg/L after the reaction, indicating that the CsPbBr_3_/Bi_2_WO_6_ composite photocatalyst is ideal for mineralizing PAHs. The products mainly consisted of monocyclic aromatic hydrocarbons with low toxicity and carbon oxide, as listed in Table S3. Although increasing the ratio of CsPbBr_3_ in the composites enhances the light absorption due to the outstanding light harvesting capacity of perovskite, overloading leads to aggregations resulting in a longer migration pathway for photogenerated charges. In addition, the overloading might also reduce the exposed surface area of Bi_2_WO_6_ to reactants. Consequently, increasing the perovskite ratio further is not necessary to optimize the degradation rate. To the best of our knowledge, there is no previous research on the photocatalytic degradation of PAHs in tobacco tar. Thus, we compared our optimal sample (over 96% degradation in 50 min) with that for PAH removal in water treatment, such as TiO_2_ (over 99% in 24 h) [49], ZnO/TiO_2_ (98% in 2 h) [50], TiO_2_ (over 66% in 39 min) [51], Evonik P25 (70% in 8 h) [52] and Pr/TiO_2_ (over 99% in 3 h) [53].

**Figure 6:**
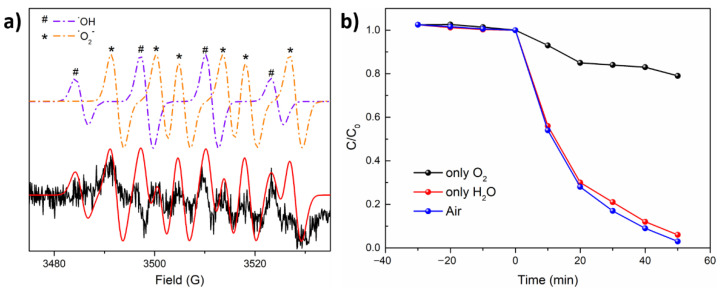
(**a**) EPR spectra of CsPbBr_3_/Bi_2_WO_6_ sample saturated with air under illumination for 5 min and 5,5-dimethyl-pyrroline N-oxide (DMPO) was used as trap agent; (**b**) photocatalytic degradation rate of 15 wt% CsPbBr_3_/Bi_2_WO_6_ sample when saturated with different gases.

To investigate the photodegradation process, electron paramagnetic resonance (EPR) spectra were employed to confirm the generated reactive radicals under simulated solar light. The EPR signals of hydroxyl and superoxide radicals (Figure 6a) were recorded [38,54,55] after illumination for 5 min as these two radicals are typically considered responsible for the high degradation rate of PAHs. Control experiments were carried out to conf irm the respective influence of these radicals on the photodegradation. To this end, one sample was degassed by Ar and then saturated with O_2_ to eliminate the source of hydroxyl generation, another sample was degassed with Ar and then 20 μL H_2_O was added to inhibit the superoxide generation. Compared to the sample saturated with air, the degradation rate dramatically reduced in the absence of a source for hydroxyl generation (Figure 6b). In contrast, the sample showed a dramatically decreased degradation rate when the source of superoxide radicals was excluded. These results suggested that the hydroxyl radicals with oxidation reactivity were predominant in the degradation of PHAs, and degradation benefits from the presence of water and oxygen. Aside from photocatalytic activity, the stability of a photocatalyst is an important criterion. As shown in Figure S3, the photocatalytic activity only decreased by 7% after six consecutive cycles, suggesting that CsPbBr_3_/Bi_2_WO_6_ photocatalyst has high stability and reusability. The XRD spectra in Figure S4 shows that the diffraction peaks of the material’s crystal structure did not change significantly after six cycles. In addition, no trace of Pd ions was found after the reaction, which can be attributed to the stable nature of CsPbBr_3_ in apolar solvent.

## 4. Photocatalytic Mechanism

Based on the Tauc plots shown in Figure S5a,b, the band gap energy (E_g_) of the Bi_2_WO_6_ and CsPbBr_3_ samples were measured to be 2.71 eV, and 2.39 eV from the linear part of (αhv)^2^ − (hv) curves. The VB values (E_VB_) and CB values (E_CB_) for a semiconductor can be calculated according to the following equations:

E_CB_ = E_VB_ − E_g_ (1)

E_VB_ = χ − E_e_ + 0.5E_g_ (2)

**Figure 7:**
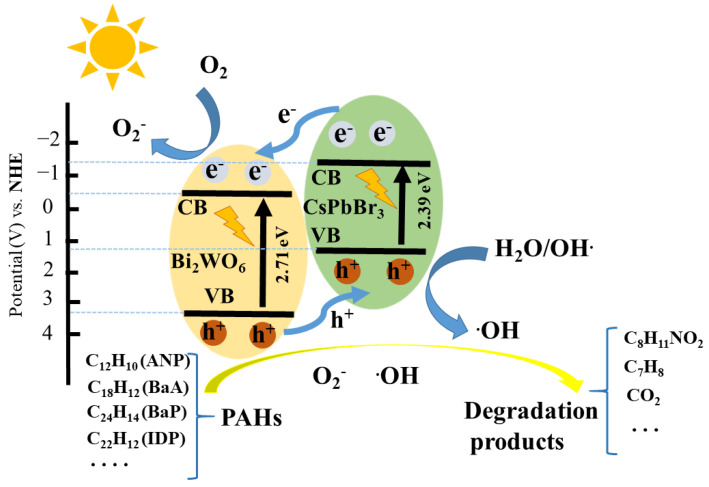
Schematic photocatalytic reaction process and charge transfer of the CsPbBr_3_/Bi_2_WO_6_ composite catalyst under solar light irradiation.

The electronegativity (χ) of Bi_2_WO_6_ and CsPbBr_3_ is 6.36 eV and 4.38 eV, respectively, according to previous reports [56,57]. E_e_ was 4.5 eV, which is the energy of free electrons on a hydrogen atom. Thus, we can conclude that the E_VB_ of Bi_2_WO_6_ and CsPbBr_3_ was 3.25 and 1.075 eV while their associated E_CB_ was 0.505 eV and −1.315 eV, respectively, versus normal hydrogen electrode (NHE). On the basis of the above results, a possible photocatalytic mechanism for degradation of PAHs was proposed (Figure 7). The type II heterojunction was formed at the interfaces between CsPbBr_3_ and Bi_2_WO_6_. Under illumination, both CsPbBr_3_ and Bi_2_WO_6_ were excited to produce photogenerated electrons and holes. Then, to achieve Fermi level balance, the conduction electrons of CsPbBr_3_ were transferred to the conduction band of Bi_2_WO_6_ under the potential difference of the band energy. At the same time, the valence holes of Bi_2_WO_6_ were transferred to the VB of CsPbBr_3_. Finally, both CsPbBr_3_ and Bi_2_WO_6_ have improved charge separation efficiency, and hence increased activity for oxidation and reduction, respectively. The reserved holes can oxide water molecules or hydroxyl ions to produce hydroxyl radicals. Meanwhile, the reserved electrons can reduce O_2_ to yield superoxide radicals. Finally, according to the EPR spectra results, PAHs were degraded into octopamine, carbon dioxide, toluene and other substances under the joint action of superoxide and hydroxyl radicals. Of the two active radicals, hydroxyl radicals were predominant in the photocatalytic reaction.

## 5. Conclusions

In this research, CsPbBr_3_ QDs decorated Bi_2_WO_6_ was successfully prepared by using a hydrothermal method. The sample was used as a photocatalyst to investigate its novel application for photodegradation of tobacco tar under visible radiation. The CsPbBr_3_/Bi_2_WO_6_ presented outstanding activity compared to classic metal oxides catalysts, and the degradation products were mainly low toxic compounds. The findings of this work may open a promising avenue for producing high-efficiency photocatalysts with special heterostructures for removing organic contaminants from used tobacco tar, and could potentially be applied to novel cigarette filters in the future.

## Figures and Tables

**Table 1:** Relative atomic content of pure Bi_2_WO_6_ and 15 wt% CsPbBr_3_/Bi_2_WO_6_ samples.

Sample	Bi4f/%	W4f/%	O1s/%	C1s/%	Br3d/%	Pb4f/%	Cs4d/%
Bi_2_WO_6_	1.62	2.53	24.04	71.8	0	0	0
CsPbBr_3_/Bi_2_WO_6_	2.21	2.15	21.7	72.74	0.96	0.09	0.16

**Table 2:** Hall effect measurement of pure Bi_2_WO_6_ and 15 wt% CsPbBr_3_/Bi_2_WO_6_ samples.

Sample	Carrier Density (cm^−3^)	Hall Mobility (cm^2^V^−1^s^−1^)	p/n Type
Bi_2_WO_6_	2.738 × 10^11^	25.316	n
CsPbBr_3_/Bi_2_WO_6_	5.154 × 10^14^	4.672	p

**Table 3:** The specific surface of Bi_2_WO_6_ and CsPbBr_3_/Bi_2_WO_6_.

Catalyst	BET Surface Area (m^2^/g)	t-Plot External Surface Area (m^2^/g)
CsPbBr_3_/Bi_2_WO_6_	9.88	9.70
Bi_2_WO_6_	5.04	5.34

## Data Availability

Not applicable.

## Supplementary Materials

The following are available online at https://www.mdpi.com/article/10.3390/nano11092422/s1, Figure S1. (a) adsorption/desorption isotherms of pure Bi_2_WO_6_ and CsPbBr_3_/Bi_2_WO_6_; (b) the TGA curves of pure Bi_2_WO_6_ and CsPbBr_3_/Bi_2_WO_6_; Figure S2. Degradation rate of PAHs using 15 wt% CsPbBr_3_/Bi_2_WO_6_ composite photocatalyst with different CsPbBr_3_ weight ratios; Figure S3. Degradation rate of PAHs using 15 wt% CsPbBr3/Bi2WO6 composite photocatalyst with air in different reaction cycles; Figure S4. XRD patterns of 15 wt% CsPbBr_3_/Bi_2_WO_6_ before reaction and after six reactions; Figure S5. Tauc plots of the Bi_2_WO_6_ (a) and CsPbBr_3_ (b); Table S1. The total organic carbon (TOC) content of PAHs with 15 wt% CsPbBr_3_/Bi_2_WO_6_ nanocomposite; Table S2. The main substrates from tobacco tar identified by GC-MS; Table S3. The main products from the photodegradation of tobacco tar. Noted that there are side-products with low concentration.
