# Supplementary material for: Photocatalytic Degradation of Tobacco Tar Using CsPbBr3 Quantum Dots Modified Bi2WO6 Composite Photocatalyst
Source: Nanomaterials (Basel). 2021 Sep 17;11(9):2422. doi: 10.3390/nano11092422 (PMC8472219; doi:10.3390/nano11092422)
Supplement: Supplementary file 1 [file nanomaterials-11-02422-s001.zip › nanomaterials-1327522-supplementary.pdf]

Photocatalytic degradation of tobacco tar using $CsPbBr_3$ Quantum Dots Modified Bi_2WO_6 Composite Photocatalyst

Runda Huang[1], Menglong Zhang[2*], Zhaoqiang Zheng[1], Kunqiang Wang[3], Xiao Liu[3*], Qizan Chen[1] and Dongxiang Luo[13*]

[1]School of Materials and Energy, Guangdong University of Technology 510006, PR China；

[2]Institute of Semiconductors, South China Normal University, Guangzhou, Guangdong 510631, PR China;

[3]School of Chemistry and Chemical Engineering/Institute of Clean Energy and Materials/Guangzhou Key Laboratory for Clean Energy and Materials/Huangpu Hydrogen Innovation Center, Guangzhou University, Guangzhou 510006, PR China

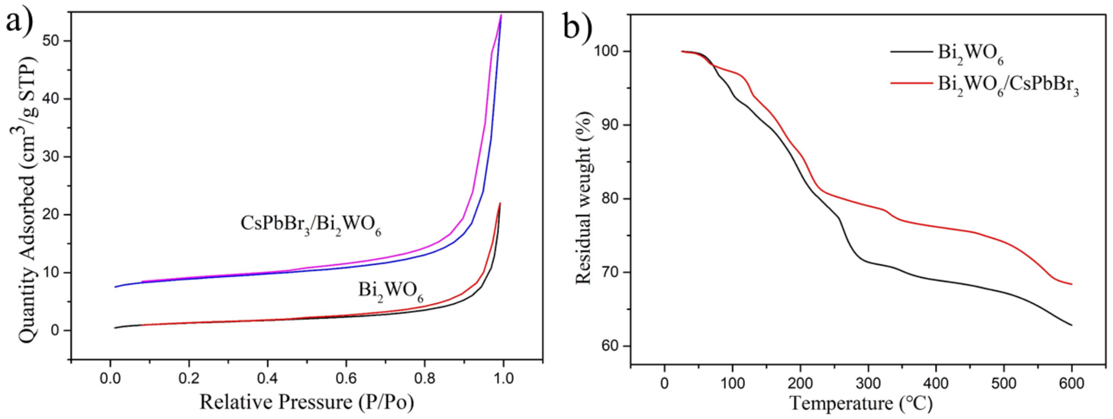

Figure S1. a) adsorption/desorption isotherms of pure Bi_2WO_6 and $CsPbBr_3/Bi_2WO_6$; b) the TGA curves of pure Bi_2WO_6 and $CsPbBr_3/Bi_2WO_6$.

Table S1. The total organic carbon (TOC) content of PAHs with 15 wt% $CsPbBr_3/Bi_2WO_6$ nanocomposite.

Time	TOC(mg/L)	TC (mg/L)	IC (mg/L)
Before reaction	5.6	6.0513	0.4513
After reaction	2.3	2.433	0.133

Table S2. The main substrates from tobacco tar identified by GC-MS

PAHs	Abbreviation	Concentration (μg/mL)
Acenaphthene	ANP	1.28
Benzo(a)anthracene	BaA	2.36
Benzo(b)fluoranthene	BbF	1.47
Benzo(k)fluoranthene	BkF	1.68
Benzo(a) perylene	BaP	2.15
Benzo(g,h,i) perylene	BgA	1.77
Fluorene	FLU	1.45
Fluoranthene	FLT	1.89
Indeno(1,2,3-cd) Pyrene	IDP	1.92
Phenanthrene	PHT	1.12

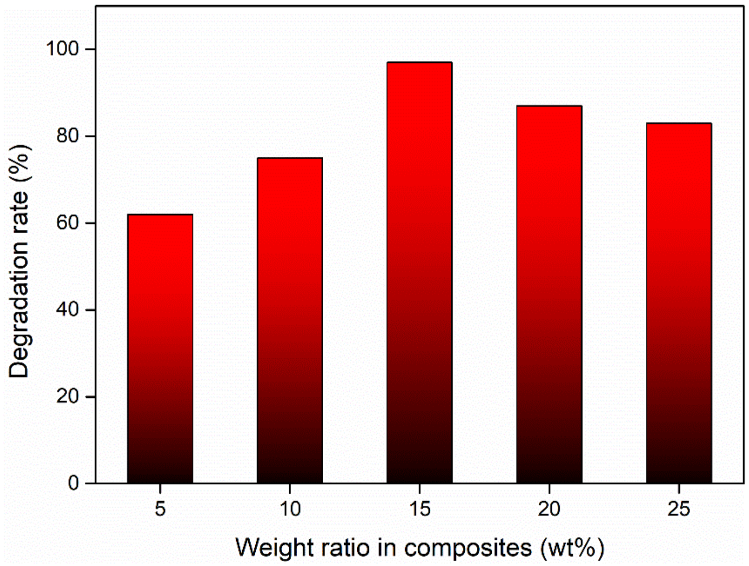

Figure S2. Degradation rate of PAHs using 15 wt% $CsPbBr_3/Bi_2WO_6$ composite photocatalyst with different $CsPbBr_3$ weight ratios.

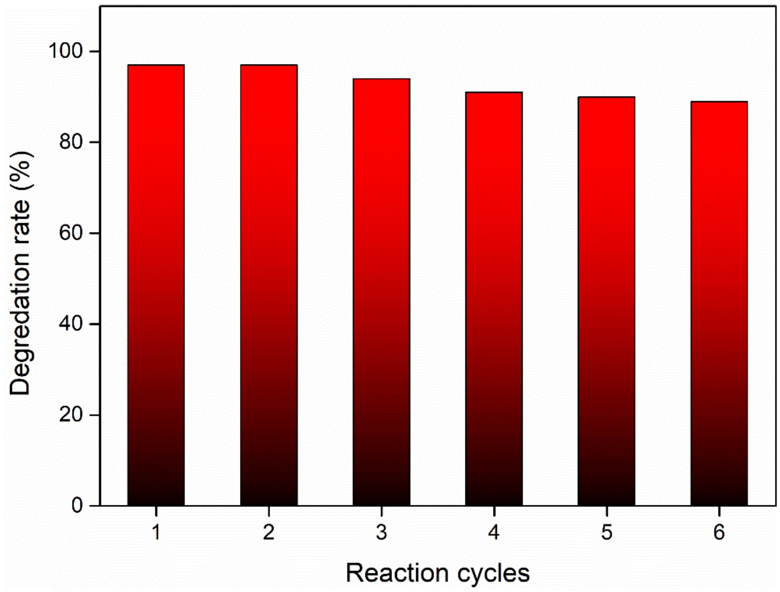

Figure S3. Degradation rate of PAHs using 15 wt% $CsPbBr_3/Bi_2WO_6$ composite photocatalyst with air in different reaction cycles.

Table S3. The main products from the photodegradation of tobacco tar. Noted that there are side-products with low concentration.

Product names	Chemical Structure	Molar percentage (%)
Benzoquinone,q	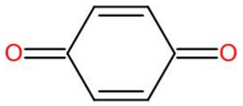	12.6
Octopamine	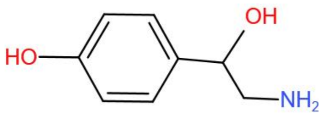	25.7
Benzoquinone 2,5 alkyl, 1,4 dione	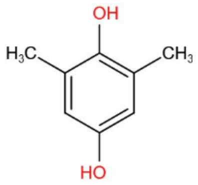	18.2
2-amino toluene	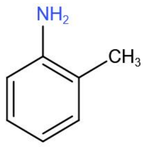	22.4
Carbon dioxide	O=C=O	21.1

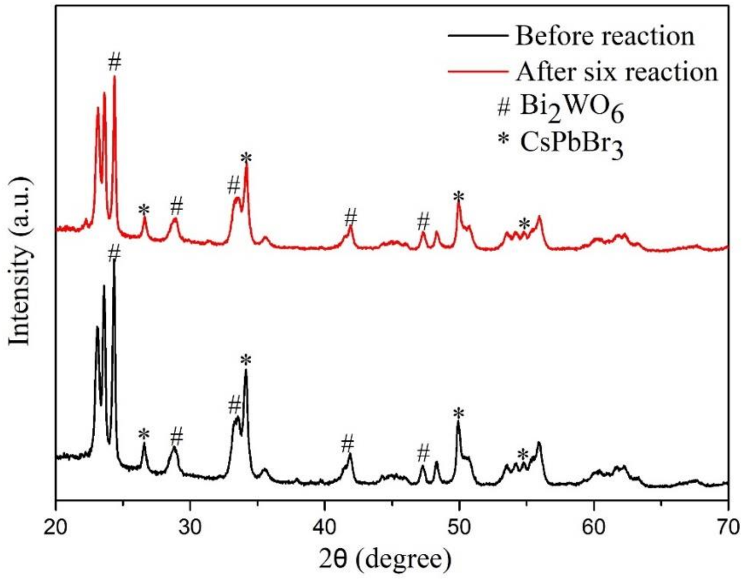

Figure S4. XRD patterns of 15 wt% $CsPbBr_3/Bi_2WO_6$ before reaction and after six reactions

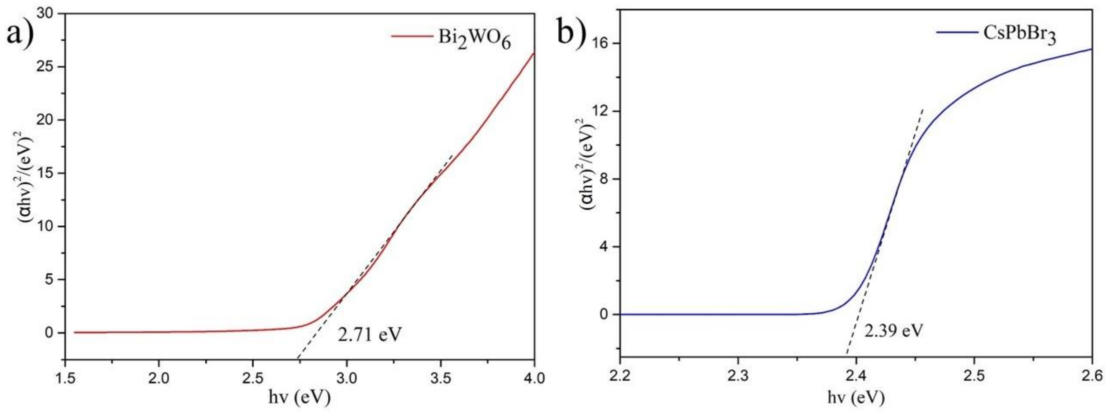

Figure S5. Tauc plots of the Bi_2WO_6 (a) and $CsPbBr_3$ (b).
